# Bee Updated: Current Knowledge on Bee Venom and Bee Envenoming Therapy

**DOI:** 10.3389/fimmu.2019.02090

**Published:** 2019-09-06

**Authors:** Manuela B. Pucca, Felipe A. Cerni, Isadora S. Oliveira, Timothy P. Jenkins, Lídia Argemí, Christoffer V. Sørensen, Shirin Ahmadi, José E. Barbosa, Andreas H. Laustsen

**Affiliations:** ^1^Medical School, Federal University of Roraima, Boa Vista, Brazil; ^2^Department of Biotechnology and Biomedicine, Technical University of Denmark, Lyngby, Denmark; ^3^Department of Physics and Chemistry, School of Pharmaceutical Sciences of Ribeirão Preto, University of São Paulo, Ribeirão Preto, Brazil; ^4^Department of Biotechnology and Biosafety, Eskişehir Osmangazi University, Eskişehir, Turkey; ^5^Department of Biochemistry and Immunology, Medical School of Ribeirão Preto, University of São Paulo, Ribeirão Preto, Brazil

**Keywords:** bee antivenom, bee allergy, bee envenoming, bee therapy, bee toxins, bee venom

## Abstract

Honey bees can be found all around the world and fulfill key pollination roles within their natural ecosystems, as well as in agriculture. Most species are typically docile, and most interactions between humans and bees are unproblematic, despite their ability to inject a complex venom into their victims as a defensive mechanism. Nevertheless, incidences of bee stings have been on the rise since the accidental release of Africanized bees to Brazil in 1956 and their subsequent spread across the Americas. These bee hybrids are more aggressive and are prone to attack, presenting a significant healthcare burden to the countries they have colonized. To date, treatment of such stings typically focuses on controlling potential allergic reactions, as no specific antivenoms against bee venom currently exist. Researchers have investigated the possibility of developing bee antivenoms, but this has been complicated by the very low immunogenicity of the key bee toxins, which fail to induce a strong antibody response in the immunized animals. However, with current cutting-edge technologies, such as phage display, alongside the rise of monoclonal antibody therapeutics, the development of a recombinant bee antivenom is achievable, and promising results towards this goal have been reported in recent years. Here, current knowledge on the venom biology of Africanized bees and current treatment options against bee envenoming are reviewed. Additionally, recent developments within next-generation bee antivenoms are presented and discussed.

## Introduction

Bees are economically beneficial insects whose existence dates back to the Cretaceous period during the Mesozoic era (1). Bees have provided several products to humans, such as honey, beeswax, pollen, royal jelly, and propolis (2). They also pollinate a wide variety of agricultural crops (3). Although bees are extremely beneficial to crops and humans, they do present a danger due to their ability to inflict painful and toxic stings (4). Fortunately, most honey bees are not aggressive towards humans and only attack when they feel threatened. However, due to the human introduction of the Africanized bee, a hybrid with highly aggressive behavior, massive bee sting attacks have markedly increased and are now endemic in most of the Americas (excluding Chile and Canada) (5). Standardized medical approaches exist for handling cases, where victims allergic to venom components are stung by bees, or where milder envenomings are caused by only a few bee stings. Yet, no antivenom exists for treating severe bee envenomings. The underlying reason for this derives from the low immunogenicity of bee venom proteins (e.g., melittin), which hinders successful immunization of production animals to yield high antibody titers in their plasma and, consequently, complicates the development of a bee antivenom significantly (6). To develop a treatment against severe bee envenoming, the design of an effective antivenom is a necessity. Here, current knowledge on bee biology, spreading of Africanized bee hybrids in the Americas, and the bee venom apparatus and toxins are reviewed, and a discussion on current and next-generation treatments of bee envenomings is provided.

## Bee Species, Behavior, and Epidemiology

Honey bees (*Apis* species) are social insects that live in well-organized communities and are very important to a significant proportion of the world economy due to the key role they fulfill as pollinators in agriculture (7). However, over the past decade, they have received increasing attention due to another physiological feature: their ability to deliver a venomous sting (8). The bee species predominantly responsible for human envenomings are *Apis mellifera mellifera* (*A. m. mellifera*) and *A. m. ligustica* in Europe, and *A. m. scutellata* in Africa (8).

Bee stings are not a novel phenomenon. In fact, significant exposure of humans to bee stings dates back over 7,000 years, when humans started to manage bee populations by providing them with artificial hives to enable an efficient harvest of their honey and wax, or for pollination purposes (9, 10). Despite significant breeding efforts, honey bees remain to be successfully domesticated, and a reduction in additive genetic variance, fixation of alleles associated with traits of economic importance, increased tameness, and the development of breed-specific characteristics amongst other properties have not been reported (11). In fact, targeted breeding appears to have increased rather than decreased genetic diversity (12).

**Figure 1:**
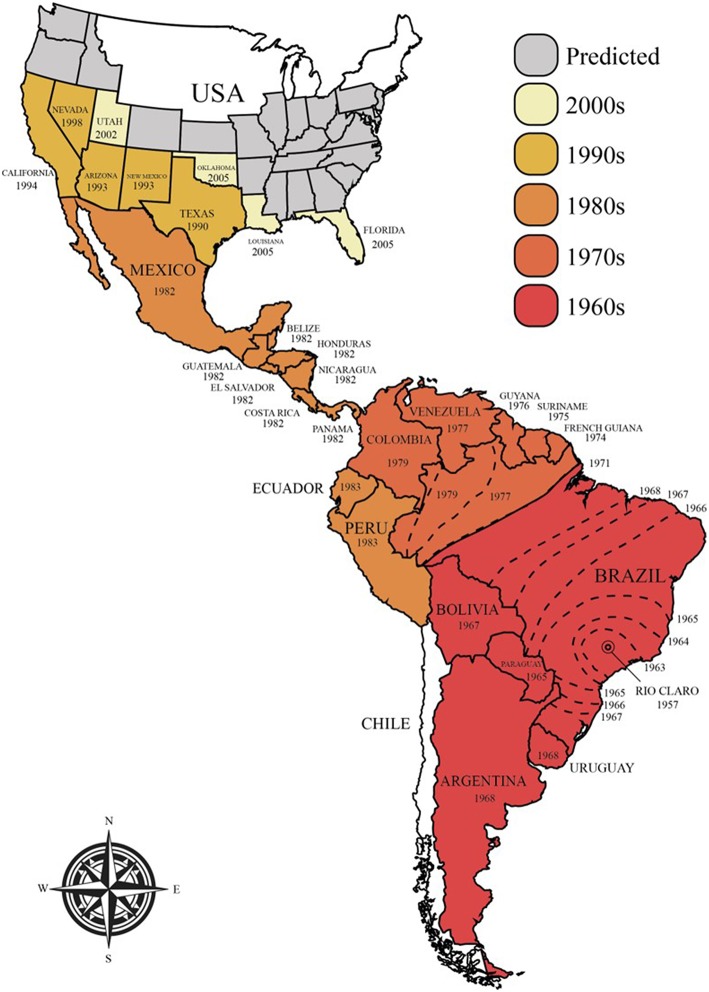
Current and predicted future spread of Africanized honey bees in the Americas.

The majority of commercial honey bee populations are derived from Europe, although they from an evolutionary perspective originated from Africa and were introduced to Europe through two independent migration events (13). In the 1620s, European honey bees (*A. m. mellifera*) were successfully introduced to North America for pollination and honey production. Later, in 1822, they were introduced to Australia (14). Attempts to replicate the original successes from North America and Australia failed in 1839 in Brazil and in other tropical regions (15, 16). This failure was believed to stem from the very different climates on both continents. New attempts were made in 1955 involving the African honey bee (*A. m. scutellata*), which was crossbred with honey bees of European descent to create a hybrid species that would better thrive in tropical environments and would produce large quantities of high quality honey (15–18). In the subsequent year, however, 26 queens and their swarms of Africanized (hybrid) honey bees escaped the laboratory and invaded large parts of the Americas, expanding 300–500 km per year (Figure 1) (15–17). The bees reached Mexico in 1986, the USA in 1990 (Texas), and have since spread into many states, including California, Arizona, Utah, New Mexico, Oklahoma, Louisiana, Arkansas, Alabama, and Florida (16, 19–22). Although climate limitations, particularly cold winters, have significantly slowed down the spread of these hybrid bees and currently restrict the range of their habitat, they are still believed to be able to colonize North America, where the harsh winter will be their only natural barrier. This range is likely to expand with global increases in temperatures (16, 23).

The success of Africanized honey bees in the Americas has been attributed to a combination of ecological and genetic factors that have provided them with increased fitness compared to the resident pollinators (15, 16). Examples include higher reproductive rates, a shorter developmental cycle (i.e., the worker bees take 19–20 days, instead of 21, and queens take 14 days, instead of 16 to mature), higher drone production/abundance, higher absconding rates (i.e., forced colony relocation in case of food scarcity) and higher swarming rates (natural colony expansion and reproduction; 6–12 times per year in case of food abundance), lower honey-storing needs, disease resistance, and decreased selectivity when choosing nest sites (15, 24–26). Furthermore, Africanized bees are significantly more defensive than other bees. This is manifested in their propensity to attack with little stimulation, increased numbers of bees that co-attack at a greater distance to the hive than usual, their pronounced insistence to chase intruders for a longer period of time, and their release of putatively larger volumes of venom (15, 16). These characteristics have led to them being commonly known as “killer bees”.

The increased aggression of these bee hybrids is thought to cause significant ongoing livestock losses and human health issues, yet there is a scarcity of reliable information on the frequency of massive stinging events and severe envenomings (8, 27, 28). This lack of data is likely because the majority of bee stings are of minor medical importance and the stung individuals do not seek medical care (8, 27). Furthermore, few governmental agencies collect data on sting frequencies (8), and often group all animal bites and stings together, in the medical records from the emergency departments (29). In the US, for instance, the annual report of the American Association of Poison Control Centers stated that 41,850 animal bites/stings and four deaths occurred in 2017, yet the lack of specificity of the data makes it impossible to attribute a certain number of cases to bee envenomings (29). Indeed, although the recent report from the Centers for Disease Control and Prevention (CDC) shows that accidents and deaths by stinging insects increased over the last 5 years (annual average of 62 deaths), the report combines accidents within hornets, wasps, and bees together (30). However, though one exemplary report for envenomings by terrestrial animals in Brazil exists, where data was collected over the course of 12 years (8, 27). The study found that a total of 1,192,667 envenomings were recorded between 2001 and 2012, of which 66,283 (5.6%) could be attributed to bees. Notably, bee stings had the second highest case fatality rate (0.33%; 216 deaths), with only snakebites exceeding them (0.43%; 3,394 deaths) (27). The study showed that case fatality rates did not appear to undergo any significant fluctuations between 2001 and 2012 (27). Due to the significant incidence of bee stings in Brazil, it is likely that similar public health issues exist in other countries in the Americas with large numbers of Africanized honey bee colonies (27, 28). In fact, since their arrival in the USA, there have been several reports of deaths after Africanized bee swarm attacks (31). Taken together, the significant number of bee stings and the relatively high fatality rates of these stings suggest that there is a growing medical need for innovative treatment options, such as specific bee antivenoms to address severe envenomings. However, the financial prospects of developing antivenom products for the market are currently unknown and difficult to predict.

## Bee Sting and Venom

**Figure 2:**
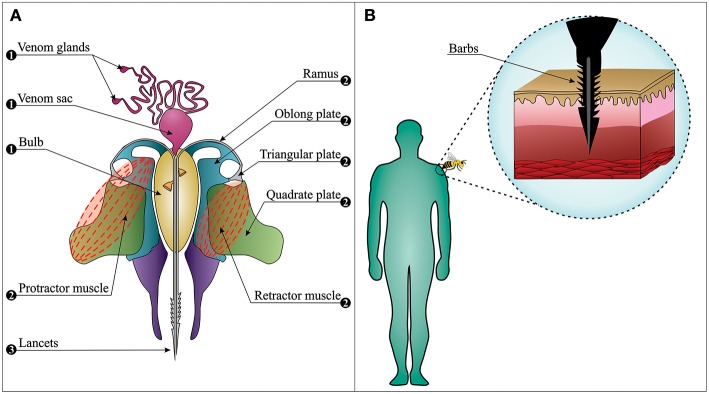
The bee sting apparatus. **(A)** The venom apparatus consists of three functionally distinct parts: (1) The venom-related part is composed of a venom sac, two venom glands, and a bulb. (2) The motor part is composed of muscles, plates, and ramus on each side. (3) The piercing part is composed of two lancets and a stylet (note: the stylet cannot be observed in this figure since the longitudinal section has passed from the middle of the venom canal that leaves the stylet on the upper section). **(B)** Barbs anchor the stinger into the skin, from where the stinger cannot be retracted when the bee escapes (i.e., sting autotomy).

The bee sting apparatus exhibits three functionally distinct parts; the motor part, the piercing part, and the venom-related part (Figure 2A) (32–34). In the piercing part, stylet, and lancets have important roles. They are covered by tetrahedron-shaped barbs, which are distributed in a spiral right-handed manner. This specific type of distribution plays a fundamental role in the helically clockwise rotation of the sting during the penetration of the stinger into the wound and reduces the penetration force (35). These barbs make it almost impossible for the bee to retract its stinger from the elastic flesh of mammals when escaping (Figure 2B). This situation may easily lead to sting autotomy, where the sting apparatus and its associated muscles are separated from the rest of the abdomen upon the bee's escape from the victim (36).

Contrary to popular belief, worker bees stay alive for 18–114 h after the sting autotomization and continue playing their role as defenders (37). When the bee escapes, its autotomized sting continues to embed itself into the wound over a period of ~30 s (38), and venom can still be delivered. It is noteworthy that at least 90% of the venom sac content is delivered within the first 20 s after the stinging event (38), and removal of the stinger (see section Bee Envenomings: Clinical Manifestations) 1 min after this event is unlikely to reduce venom-induced toxicity. On average, 140–150 μg of venom is delivered in a stinging event, and the median lethal dose (LD_50_) of bee venom varies between 2.8 and 3.5 mg of venom per kg of human body weight (38–41). It can thus be speculated that a non-allergic person weighing 60–70 kg has a 50% chance of death upon being stung by 1,000–1,500 bees, although deaths caused by only 200–500 stings have also been reported (38, 42). Indeed, the severity of the envenoming is determined by victim age, body weight, number of stings, and individual characteristics of the victim (immune status, comorbidities, and previous sensitization) (43).

Bee venom is a complex mixture of compounds, which include proteins, peptides, amino acids, phospholipids, sugars, biogenic amines, volatile compounds, pheromones, and a high quantity of water (>80%) (44–46). The composition of bee venom has already been elucidated by omics techniques (47–49) and by fractionation of the venom (50–53). In this review, only components with important clinical and therapeutic effects, and with enough literature support, will be detailed, while other bee venom compounds are only listed in Table 1. It is important to emphasize that bees are insects from the Hymenoptera order, which includes wasps (80). Therefore, bee venoms contain some of the same compounds as wasp venoms, such as adrenaline, dopamine, histamine, hyaluronidase, noradrenaline, phospholipases A_2_ (PLA_2_s), phospholipases B (PLBs), and serotonin (81), while only bee venoms contain apamin (82), melittin (50), and mast cell-degranulating peptide (MCD) (83, 84).

**Table 1:** Bee venom compounds.

**Name (others)**	**Mass (Da)**	**Access (uniprot)**	**% of dryed venom^**#**^**	**References**
α-Glucosidase	65,565	Q17058	0.6	(54, 55)
Acid phosphatase (Api m 3)	45,389	Q5BLY5	1	(56)
Adolapin	11,500 11,092	–	0.1–0.8	(57)
Apamin	5,223	P01500	1–3	(58)
Api m 6.01	7,190	P83563^*^	–	(59)
Api m 6.02	7,400			
Api m 6.03	7,598			
Api m 6.04	7,808			
Cardiopep	1,940	–	0.7	(60)
Dipeptidylpeptidase IV (Api m 5)	87,937	B2D0J4	–	(61)
Hyaluronidase (Api m 2)	44,260	Q08169	1–3	(62)
Icarapin (Api m 10)	24,819	Q5EF78	–	(63, 64)
MRJP (1–5)	49,000	O18330	–	(65)
	87,000			
		O77061		
		Q17060		
		Q17061		
		O97432		
MRJP9 (Api m 11.0101)	48,518	Q4ZJX1	–	(66)
MCD (Peptide 401)	5,781	P01499	1–3	(67)
Melittin	2,846	P01501^*^	50–60	(68–70)
Melittin-S	2,830		1–2	
Synthetic melittin	–		–	
Melittin-F	2,208	–	0.01	(71)
Minimine	6,000	–	2–3	(72)
PLA_2_ (Api m 1)	19,058	P00630	10–12	(73, 74)
PLB (Lysophospholipase)	–	–	–	(75)
Procamine	<1,000	–	1.4	(44)
Secapin	8,664	P02852	1–2	(71)
Secapin-1	2,822	–	1	(76)
Secapin-2	2,872	–	–	(77)
Serine proteases (Api m 7)	39,000	–	–	(78)
Tertiapin	2,459	P56587	0.1	(79)

*MRJPs, Major Royal Jelly Proteins; MCD, Mast Cell-Degranulating peptide; PLA_2_, phospholipase A_2_; PLB, phospholipase B*.

* *Isoforms are represented by the same entry in the UniprotKB due to the small differences in their amino acid sequence*.

# *Dried venom excludes volatile compounds*.

**Figure 3:**
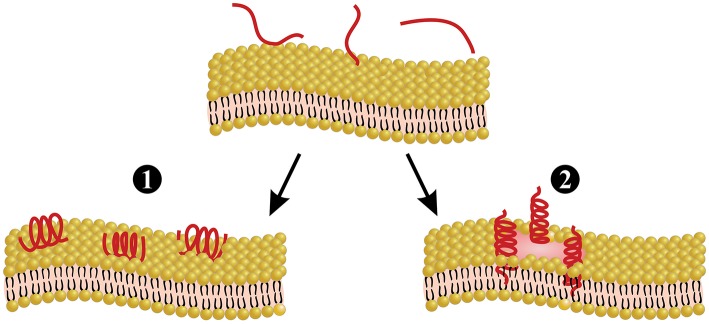
Melittin-induced pore formation model. Melittin can bind to the membrane either in a parallel orientation (1) or a perpendicular orientation (2). The perpendicular orientation induces pore formation, whereas the parallel orientation is inactive. Parallel orientation has also been hypothesized to protect the membrane, since this prevents other melittin molecules from forming pores. Figure adapted from van den Bogaart et al. (90).

Melittin is the main and most toxic compound in bee venom, constituting 50–60% of the whole venom (85). Melittin only induces minor allergic reactions (86), but causes the majority of the pain associated with bee stings (4), which is induced through direct and indirect actions on primary nociceptor cells. The direct action is caused by melittin activation of thermal nociceptor transient receptor potential vanilloid 1 (TRPV1) via the PLA_2_ cascade pathway, resulting in sensitization of the primary nociceptors (87–89). The indirect action is based on the pore-forming actions of melittin (Figure 3), which allows for the release of pain-inducing substances such as H^+^, adenosine triphosphate (ATP), and 5-hydroxytryptamine (5-HT) from mast cells, as well as melittin causes tissue damage, resulting in activation of the pain receptors. Pore formation induced by melittin can also release mediators, such as histamine, bradykinin, and ATP, which activate G-protein-coupled receptors (GPCRs), resulting in the phosphorylation of phospholipase C (PLC). PLC cleaves phosphatidylinositol 4,5-bisphosphateintodiacylglycerol (DAG) and inositol 1,4,5-trisphosphate (IP3). DAG is an endogenous activator of transient receptor potential canonical (TRPC) channels, resulting in the indirect excitation of primary nociceptive neurons (i.e., pain). A more detailed overview of the pain-inducing mechanism of melittin can be found elsewhere (4). Melittin is classified as a lytic peptide that is able to destroy membrane phospholipids, which include erythrocytes, leading to hemolysis (85, 91). This action may derive from conformational modification, where melittin molecules have been proposed to bind perpendicularly to membranes forming a pore (90) (Figure 3). Furthermore, melittin can increase the activity of PLA_2_s (92). As an example, PLA_2_ activity was tested with and without melittin on lecithin liposomes, and it was observed that PLA_2_ activity was 5-fold higher in the presence of melittin (92). Different isoforms of melittin can be found in bee venom, such as melittin-S and melittin-F. However, these exist only in low abundance (68, 71). Melittin has been demonstrated to have antimicrobial activity *in vitro* and *in vivo* (91), anti-inflammatory effects *in vitro* (93), antiviral activity *in vitro* (94), and anti-cancer effects *in vitro* and *in vivo* (95). Finally, it is worth mentioning that melittin can be chemically synthesized to obtain high amounts of the peptide (69, 96, 97).

Phospholipase A_2_ (PLA_2_) is the second most abundant compound (10–12%) and the most allergenic and immunogenic protein in bee venom (83). Alone, PLA_2_ is a non-toxic protein (44, 83), but when PLA_2_ forms a complex with melittin, called bee hemolytic factor, it cleaves cellular membrane phospholipids (98). *In vitro*, bee PLA_2_ possesses several activities, such as trypanocidal and antibacterial effects (99, 100), neuronal protection caused by prion proteins (101), and anti-tumor properties (102). Moreover, bee PLA_2_ was able to decrease hepatotoxicity caused by acetaminophen in mice (103). Phospholipase B (PLB) has also been reported to be present in bee venom (75). PLB exhibits both PLA_1_ and PLA_2_ activity, being responsible for cleaving phospholipids on sn-1 and sn-2 position of acyl chains (104), which enhances PLA_2_ activity (46). Notably, PLBs are also important components of snake venoms (46, 75, 105, 106).

Apamin is another important peptide in bee venom, which comprises 1–3% of crude venom and is able to allosterically and selectively inhibit Ca^2+^-dependent K^+^ channels (SK channels), found in the central nervous system (CNS) (81, 107, 108). Only SK2 and SK3 channels are known to be sensitive to apamin, and when blocked, there is a decrease of the delayed hyperpolarization of cells, which results in increased continuous firing of neurons in the mesencephalon and cerebellum, elevating cell sensitivity to excitatory inputs (107, 109). Moreover, apamin is able to activate inhibitory muscarinic receptors of motor nerve terminals (i.e., reducing neuromuscular transmission), which has been experimentally explored as a potential treatment against diseases presenting high muscle excitability (110), such as Parkinson's disease (111), learning deficit disorder (112), and other disabilities (81, 113).

Hyaluronidase is an enzyme found in bee venom (1–3%), as well as many other animal venoms (114–117). Hyaluronidase is responsible for fast distribution of toxins, also known as the “spreading factor,” as this enzyme cleaves hyaluronic acid from the extracellular matrix (ECM) (83, 118, 119), leading to a faster and systemic envenoming by disrupting tissues (120). In addition, hyaluronidase is considered a potent allergen in bee venom (121).

Mast cell-degranulating (MCD) peptide is also considered an important component in bee venom based on its capability to induce histamine release from mast cells, which exhibit a central role on inflammation and allergy (122). In high quantities, however, MCD presents an opposite action, where it inhibits mast cell degranulation (i.e., by inhibiting histamine release). Thus, MCD can also act as an anti-allergic molecule (123). Indeed, studies have demonstrated that MCD peptide presents anti-inflammatory activity *in vitro* and *in vivo* (124, 125).

Beside the above mentioned components, bee venom also contains amines, such as histamine and catecholamines (81). Histamine is able to increase capillary permeability, contributing to the inflammatory response, while catecholamines (i.e., noradrenaline and dopamine) enhance bee venom distribution, since they, among other functions, increase cardiac output (122).

As for other venoms (105, 126, 127), bee venom is very susceptible to variability, depending on bee age, species, social condition, geographic localization, amongst other factors (44). For instance, young worker bees (foragers/guards/nurses) have higher levels of apamin and lower levels of melittin compared to old workers (foragers/guards). In contrast, queen bees present lower levels of melittin and apamin (128) and higher levels of histamine (129). Furthermore, young bees have low levels of histamine, while, at 35-days-old, they present high levels of this molecule. Melittin reaches maximum concentration when the bee is 4-weeks-old, and then decreases during bee aging; while promelittin is most prevalent when the bees are 8–10-days-old (130). Hyaluronidase levels also vary in bee venoms. Although hyaluronidase can be detected immediately after the pupae emerge from the eggs as adult bees (i.e., eclosion), the enzyme levels increase with bee aging (131). Whilst low concentrations of PLA_2_ are found during bee eclosion, they increase gradually and reach the highest levels when the bees are 7–10-days-old (51).

Regarding venom variations among different bee species, African bees release a low amount of venom when stinging, with lower quantities of melittin and hyaluronidase, and increased amounts of PLA_2_, which can be explained by the fact that these bees possess smaller venom glands than the European bees (132–135). Additionally, seasonal changes may have an impact on bee venom content, since the seasons affect flowers and fruits, and therefore also bee feeding (104). Melittin production changes during the summer (136), while melittin-S production increases during winter, allowing mellitin-S to reach an abundance of 10% of whole venom (68).

Venom milking methods can also affect bee venom composition. Bee venom can be collected by extraction of glandular venom or by electrical stimulation, and venoms collected by these methods present differences on chromatographic profiles. Volatile components such as histamine can disappear when bee venom is collected by electrical stimulation (44, 137). Moreover, through proteomic analysis, bee venom obtained by gland extraction may have contamination of proteins from the gland tissue, so that down to only 40% of the obtained material is actual bee venom proteins. However, generally when electrical stimulation is used, more than 80% of the obtained material is venom proteins (48).

## Bee Envenomings: Clinical Manifestations

Bee envenomings can result in mild to severe clinical manifestations depending mainly on the number of stings that the victim has received. Patient age, weight, co-morbidities, and medical care can also influence the severity of an envenoming (28). Moreover, atopic individuals (e.g., individuals with asthma or allergic rhinitis) and a family history of bee sting allergy are associated with higher incidence of severe reactions (138). Typically, the clinical manifestations of bee envenoming can be divided into local inflammatory reactions (1), allergic manifestations (2), anaphylactic shock (3), and systemic toxic reactions (4) (43, 139). (1) Local inflammatory reactions are characterized by pain, swelling (edema and erythema), itching, and pruritus at the sting site. These reactions are experienced by most non-allergic individuals and are normally resolved within 24 h (39). (2) Bee sting allergic reactions are IgE-dependent and are classified as hypersensitivity type I reactions. These reactions occur about 10 min after the sting, and the symptoms can vary in severity. PLA_2_ is considered the main compound that induces IgE-sensitization of mast cells, although hyaluronidases and melittin are also considered allergens (140) (see section Bee Sting and Venom). Allergic patients can develop systemic urticaria, pruritus, angioedema, vomiting, and diarrhea (28). (3) In some cases, the allergic reactions can evolve to an anaphylactic reaction, resulting in bronchoconstriction and anaphylactic shock (39). Between 25% and 70% of patients with insect allergies exhibit systemic reactions when challenged with the allergen (i.e., bee venom) (140). Interestingly, some non-allergic individuals can also develop bee anaphylaxis due to systemic mastocytosis (141–143). Systemic mastocytosis is a heterogeneous disorder characterized by proliferation of mast cells and the extent of granulation, which is caused by mutations in the c-Kit gene (a growth factor for mast cells) (144, 145). (4) Systemic toxic reactions are characterized by direct toxic effects of the bee venom, independent of immune mechanisms, which are also known as venom volume-dependent reactions. Systemic toxic reactions are always considered severe and are caused by multiple stings (about 50 simultaneous stings). Patients suffering from systemic toxic reactions may present fatigue, dizziness, nausea, vomiting, and diarrhea, which can evolve into myocardial injury, hypertension, hepatic injury, rhabdomyolysis, hemolysis, comatose, and acute renal failure (43, 146, 147). Deaths are likely to occur when the victim has received about 500 stings, which are considered necessary to cause death by direct toxicity (42), although fewer stings (30–50) have proven fatal in children (148).

Other rare clinical manifestations have also been reported for bee stings, including peripheral neuritis (149), Fisher's syndrome (150), acute inflammatory polyradiculoneuropathy (Guillain-Barré syndrome) (151), optic neuropathy (152), septicemia (153), bilateral empyema (154), and even urticaria for a baby (12-day-old) being breastfed by its mother who had been stung by a bee (155).

## Current Treatment

**Figure 4:**
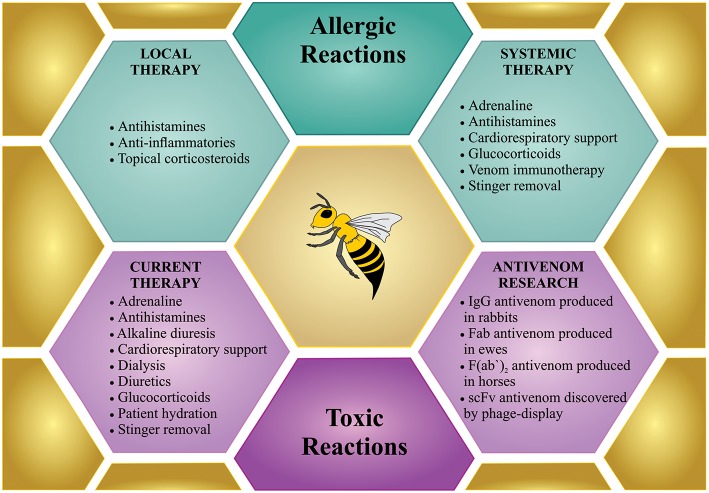
Treatment for bee sting(s). Bees incidents can involve few stings, which can cause local reactions or anaphylactic shock, which request a treatment similar to any allergic reactions (in green). However, mass stinging events can prove life-threatening via the toxic action of the venom when injected in large amounts, which demands intensive treatment (in purple). Although specific treatment is not available so far, only few antivenom researchers are working on developing new therapies against bee envenoming.

There are generally three different bee sting scenarios that require treatment. (1) Few stings on a non-sensitized person; (2) one or more stings on a hypersensitive person; and (3) massive bee envenoming by multiple stings (Figure 4).

### Treatment of a Few Stings on a Non-sensitized Person

Non-sensitized people present only a localized reaction to a bee sting. In the normal reaction to a bee sting, the skin manifests itself as an area of pain, redness, and swelling that is generally <10 cm in diameter and normally disappears within 24 h (156). Experiments conducted in guinea-pigs stung by bees, which had their tissues removed and subjected to histologic analysis, demonstrated only local marked inflammation (i.e., edema, high cellular infiltration, and necrosis) during 24 h after the sting (157). Medical assistance is unnecessary in these cases, although the use of topical corticosteroids is recommended, which produce anti-inflammatory, immunosuppressive, and anti-mitogenic effects (158). However, in the so-called large local reactions to an insect sting, the area of swelling and redness may be larger than 10 cm in diameter, and pain can persists for several days. Although this reaction is presumably of allergic origin, it is not necessarily mediated by IgE. In this situation, oral antihistamines can be prescribed (156).

### Treatment of Hypersensitivity

If a person who is hypersensitive to bee venom (i.e., an allergic person) is stung by at least one bee, immediate medical attention is required, since anaphylaxis might occur. The majority of the deaths that occur in this case are due to allergic individuals not reaching medical care fast enough (159). The preferred first line of action against anaphylaxis differs from study to study. Some studies report as first line of action to scrape off the bee stingers carefully (by avoiding to pull or squeeze the stingers, which could lead to injection of more venom) (160), although this action is mainly relevant if performed within 60 s of the stinging event, in which period the stinger ejects all its venom (39). Other studies state that the first-line of action should be to administer intramuscular adrenaline (also known as epinephrine), and only then remove the stinger (161). While these studies might disagree on whether stinger removal should be the first action followed by adrenaline, or the opposite, they do agree that the first drug to be used is intramuscular adrenaline (39, 160–162). Even a few minutes delay in the administration of adrenaline can lead to hypoxia or death. Indeed, the lack of access to emergent adrenaline plays a critical role in the mortality and morbidity for allergic patients. Thus, there has been an increased awareness of the need for adrenaline auto-injectors in public locations including schools, parks, airports, and shopping malls (163, 164).

Adrenaline acts as an α and β-agonist. Through its α-1 agonistic effect, it works as a vasoconstrictor, which prevents and relieves airway edema, hypotension, and shock. The β-1 agonistic effects of adrenaline are chronotropic and inotropic and thus increase the rate and force of cardiac contractions, while the β-2 agonistic effects of adrenaline lead to bronchodilation (162). Furthermore, the β_2_-adrenergic agonistic effects of adrenaline also increase the intracellular levels of cyclic adenosine monophosphate in mast cells, which inhibits further release of inflammatory mediators, such as histamine, leukotrienes, and prostaglandin D_2_ (165). Following the administration of adrenaline, other first-line treatments include oxygen, intravenous fluid resuscitation, and inhaled short-acting β_2_ agonists (161, 166, 167). Second-line treatment usually consists of H_1_-antihistamines, H_2_-antihistamines, and glucocorticoids, which are given as adjuvant therapy and are considered optional. The antihistamines are only recommended for the relief of cutaneous symptoms, while glucocorticoids may be effective for treating airway edema and could prevent protracted anaphylaxis symptoms (161, 166).

A preventive treatment available to allergic individuals is venom immunotherapy (VIT). VIT consists of inoculating small increasing amounts of purified venom extracts in the allergic individual over a period of time. Venom extracts were introduced in the 1970s, and since then, VIT has become increasingly popular. Several different administration regimens have been developed to shorten the time required to reach the maintenance period and to minimize side effects (168). According to the European Academy of Allergy and Clinical Immunology (EAACI), VIT can be performed using different venom products (purified and non-purified, aqueous or depot) and different treatment protocols (conventional, cluster, rush, and ultra-rush), administered by the subcutaneous or sublingual routes (169).

Effective VIT restores immunotolerance to allergens by different mechanisms: (1) desensitization of mast cells and basophils; (2) suppression of innate lymphoid cells (ILC2); (3) activation of regulatory T cells (Tregs), which increase the levels of interleukin 10 (IL-10) and transforming growth factor-β (TGF-β); (4) and immunoglobulin cell-switch to IgG4 and IgA induced by Treg cytokines (170). The decision whether to start VIT depends on an accurate diagnosis, an assessment of the person's risk of having another allergic reaction, the degree to which the allergy affects their quality of life, the person's age and comorbid medical conditions, as well as whether the person suffers from concurrent mast cell disorder. Moreover, the allergen preparation (see EAACI guidelines) and the administered dose should be taken into account to avoid adverse effects as well as to ensure therapeutic success (169). In any event, VIT needs to be performed under medical supervision due to the risk of an allergic reaction. A study found that almost one in 10 people treated with VIT had an allergic reaction to the treatment (171). However, adverse events are normally mild and, although it is recommended to reduce the allergen dose in case of systemic adverse reactions, patients should not discontinue the therapy, since VIT is effective in reducing the risk of a subsequent systemic reaction to a bee sting in 77–84% of the treated patients (169).

### Therapy Against Massive Bee Envenoming

The initial treatment against massive bee envenoming follows the same course as for a case of hypersensitivity. Allergic and systemic toxic reactions are difficult to differentiate, especially in the first minutes, and anaphylactic shock is the most immediate danger to the patient (172). However, once it is established that a hypersensitive event is not (or not only) occurring, specific treatment for massive bee envenoming is initiated. The ideal treatment against the severe toxic effects of bee venom would likely be antivenom. However, there are no specific antivenoms available, although major efforts are being made (see section Next-Generation Antivenom Therapy) (139). Patients who have more than 50 stings should be monitored, since the circulating venom toxins may persist in their body for hours or days and may have the potential to cause delayed reactions. Initially, the stung victim may be stable. Hours later though, the victim's conditions may deteriorate (28, 172, 173). Clinical monitoring should focus on levels of creatinine, serum urea nitrogen, electrolytes, and myoglobin to asses renal function and the risk of rhabdomyolysis (172, 174). Furthermore, to check for the development of acute respiratory distress syndrome and acidosis, blood pH, and oxygen levels should be monitored. If a patient shows signs of myoglobinuria, intravenous injection of sodium bicarbonate can be performed for alkalization of urine (i.e., to accelerate renal excretion). Alkaline diuresis can prevent the crystallization of myoglobin in kidney tubules, which may eventually lead to acute renal failure (172). Additionally, aggressive hydration and diuretics are often administered (139, 174). The patient can be started on either hemo or peritoneal dialysis, exchange transfusion, or plasmapheresis, to eliminate low molecular weight components of the venom, such as melittin or PLA_2_, or if acute renal failure develops (5, 172).

## Next-Generation Antivenom Therapy

One of the obstacles for producing antibodies by immunization procedures for bee envenoming therapies is the lack of immunogenicity of several of the key bee venom toxins, such as melittin. As earlier mentioned (see section Bee Sting and Venom), melittin is a cell membrane lytic factor (85, 175) with a small molecular size (5, 176, 177), random conformation (178), and very hydrophobic regions (177), resulting in low immunogenicity (6), which highly complicates the production of effective bee antivenoms, as melittin fails to induce a strong antibody response in immunized animals.

Over the past decades, several attempts to develop an effective bee envenoming therapy have been reported. In 1996, Schumacher et al. reported the first attempt to produce heterologous antibody-based bee antivenom. Here, a polyclonal mixture of immunoglobulin G (IgG) antibodies was produced by successive immunizations of rabbits with purified PLA_2_, melittin, or crude bee venom, and neutralization capacities were further assessed in mice. It was observed that the specific anti-PLA_2_ antibodies clearly reduced PLA_2_-associated toxicity, when the toxin was administrated alone to mice. In contrast, it had no significant effect on lethality once crude venom was employed. Even when a combination of anti-PLA_2_ and anti-melittin antibodies was used, crude venom lethality did not decrease in mice, although authors identified melittin-binding antibodies in the rabbit serum (176).

In 1999, Jones et al. described a different approach based on Fab (fragment antigen binding) antibody fragments. In their study, Welsh ewes were successively immunized with bee venom for the production of IgGs, which were further digested with papain to obtain Fab fragments. Using an enzyme-linked immunosorbent assay (ELISA), researchers demonstrated that the Fab-based antivenom was able to recognize melittin. In addition, using standard efficacy (ED_50_) tests *in vivo*, the researchers determined that 20.5 mg of the ovine Fab-based antiserum was required to neutralize the toxic effects and to prevent lethality in mice when the antivenom was pre-incubated with 1 mg of bee venom (179). Later, a horse antibody fragment F(ab')_2_-based antivenom, described by Santos et al. (180), was demonstrated to be efficient in neutralizing the toxic activities of bee venom *in vitro* and *in vivo*. *In vitro*, the hemolytic activity of 1 mg of bee venom was neutralized by ~50 mg of the antivenom. *In vivo*, the horse antivenom was able to completely neutralize the myotoxic effects of bee venom with an effective dose (ED_50_) of 1.11 mg/mL (mg of bee venom/mL of antivenom) (180). Also, using horse immunization, Barraviera and co-authors recently developed a horse F(ab')_2_-based antivenom with an ED_50_ of 1.25 mg/mL (181). This study also developed a protocol for phase I/II clinical trials using the generated equine antivenom (181).

The current methods for producing antivenoms are based on successive immunizations of different animals, followed by low-cost purification of the animal plasma-derived IgGs. In spite of the historical clinical success achieved with animal plasma-derived antivenoms, these envenoming therapies have a propensity to cause adverse effects due to their heterologous nature (182, 183). It has been observed that 6–59% of the snakebite patients treated with plasma-derived antivenoms experienced early-onset adverse reactions after the administration of the antivenom, whereas 5–23% of patients experienced some delayed-onset (serum sickness) reactions, with symptoms such as fever, rash, and urticaria (182, 184). These antivenom-related adverse reactions are mainly a result of the composition and quality of the antivenom, the antibody format, and/or the total amount of protein administrated to the patient (185). Plasma-derived antivenoms are the only commercially available option for envenoming therapy. However, current progress made within the fields of biotechnology and monoclonal antibodies has positively contributed to the development of experimental antivenoms based on mixtures of specific recombinant monoclonal antibodies (186–188). Although these experimental antivenoms are yet to enter the clinical setting, envenoming therapies based on recombinant monoclonal antibodies and antibody fragments are predicted to one day be brought to the market and to be economically feasible to manufacture in the future (189).

In the field of recombinant bee antivenoms, Barbosa et al. were the first to report the discovery of fully human single-chain variable fragment (scFv)-based antibodies obtained via phage display technology against melittin and PLA_2_. These toxins act synergistically, and the combination of monoclonal antibodies against these toxins may therefore find its utility in treating severe bee envenomings. Specific monoclonal antibodies against melittin and PLA_2_ were selected from a phage display library and further selected for toxin specificity via ELISA. Two different scFv clones, named A7 and C12, against PLA_2_ and melittin, respectively, were discovered. Neutralization studies demonstrated that these two clones were able to neutralize the hemolytic activity of bee venom *in vitro* at a mass to mass ratio of 3:1 (scFv:bee venom). Moreover, the same monoclonal scFvs inhibited myotoxicity and delayed mortality in mice challenged with 1.5 LD_50_ of bee venom at the same ratio (190). Later, the same researchers selected two new monoclonal scFv-based antibodies against melittin and PLA_2_ using phage display technology, named Afribumab 1 and Afribumab 2, respectively. Afribumab 1 and 2 presented the capacity to inhibit bee venom hemolysis (0.5 μg) *in vitro* at a mass to mass ratio of 1:1:1 (bee venom:Afribumab 1:Afribumab 2). Using mice challenged with 2 LD_50_s of bee venom (corresponding to 9.484 μg/g of bee venom), the combination of Afribumabs 1 and 2 with the same ratio of 1:1:1 was demonstrated to reduce edema and prolong mouse survival for more than 400 min (compared to around 100 min when the mice were only challenged with 2 LD_50_s of bee venom) (191). Combined, these studies demonstrated that phage display technology can be an effective methodology for selecting antibodies with specificity against non-immunogenic components of bee venom (e.g., melittin). Such antibodies could not have easily been generated by more traditional antibody discovery approaches relaying on animal immunization. Potentially, such monoclonal antibodies against key toxins from bee venom could be formulated into a recombinant antivenom for treating severe bee envenoming. Although the precise timing for efficacious administration of such a bee antivenom cannot be predicted due to limited knowledge on the toxicokinetics of bee venom components in human subjects, it is likely that antivenom administration should occur within 24 h, since kinetic studies in mice have demonstrated that bee venom can be detected in different organs, such as the kidneys, during this period (192).

As effective therapeutic intervention is essential for the most severe cases of massive bee envenoming, and as scientific prior art demonstrates the applicability of different biotechnological techniques and antibody discovery methodologies in this field, it is likely that significant advances within the development of recombinant antivenoms against bee envenoming will occur in the next few decades. It seems eminent that antivenom design and development approaches from the neighboring field of snakebite envenoming may be adopted in the development of next-generation bee envenoming therapies. Particularly, the investigation of the utility of different monoclonal antibody formats (including nanobodies) and possibly non-antibody-based binding proteins (such as DARPins and other emerging scaffold proteins) is warranted, as this may enable the design of recombinant antivenom products with beneficial pharmacokinetic properties, such as rapid distribution and the ability to penetrate and target toxins deep within tissues (182, 193, 194). Such investigations may also prompt the exploration of low-cost manufacturing strategies for oligoclonal antibodies (189) or formulation strategies for improved stability and extended shelf-life. Also, the use of low-cost small molecule inhibitors may be an area relevant for further research. Finally, it is even possible that efforts within the development of improved bee envenoming therapeutics may encourage research and development in the field of bee envenoming diagnostics, which may aid stratification of patients and clinical decision making.

## Final Remarks

Africanized bee attacks are considered a public health concern in Brazil, where they originated from. Other American countries have also noticed the effects of this serious threat, as these bee hybrids are currently spreading across the Americas. As a consequence, bee stings and envenomings will likely increase. The solution to this severe problem requires well-prepared medical emergency services and specific treatments against bee envenoming, such as antivenoms. To this date, only a few reports demonstrating positive results using animal immunization exist in the scientific literature, and no antivenom for treating severe bee envenomings is so far available to treating physicians. A possible explanation for the lack of commercial bee antivenoms is the difficulty of obtaining specific antibodies against key components of the bee venom, as these have low immunogenicity. Traditional methods based on successive animal immunizations therefore fail to generate high enough antibody titres for therapeutic utility. In contrast, phage display technology has proven to be a promising methodology for generating antibodies against key bee toxins with low immunogenicity. This technology may thus enable the development of effective recombinant bee antivenoms in the future (186–188). However, this technology is still a quite recent addition to the field of antivenom development, and many efforts are still needed before an effective antivenom for the treatment of severe bee envenomings will see the light of day.

## Author Contributions

FC, IO, TJ, LA, CS, and SA wrote part of the review and provided critical feedback. FC and TJ prepared figures. MP and AL designed the review, wrote part of the manuscript, and provided revisions. JB gave his valuable and professional suggestions. All authors read and approved the final manuscript.

### Conflict of Interest Statement

The authors declare that the research was conducted in the absence of any commercial or financial relationships that could be construed as a potential conflict of interest.
